# Supplementary material for: PDLIM2 in lung adenocarcinoma metastasis
Source: Neoplasia. 2026 Sep 25;81:101371. doi: 10.1016/j.neo.2026.101371 (PMC13635527; doi:10.1016/j.neo.2026.101371)
Supplement: Supplementary file 1 [file mmc1.pdf]

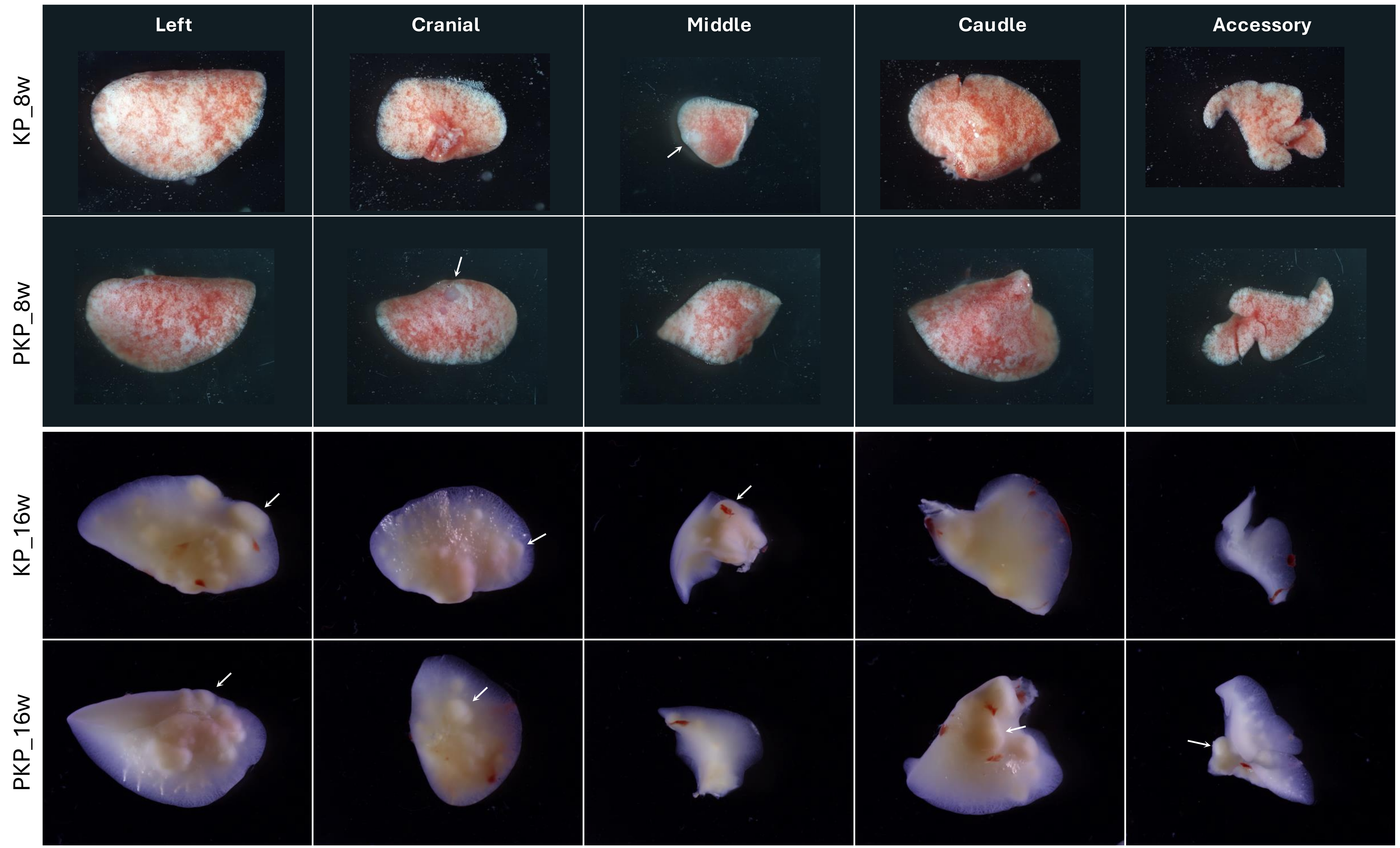


**SFig. 1. Representative lung tissue of KP and PKP mice 8 or 16 weeks post AdenoCre administration.**

Myeloid Panel

| Laser | Filter | LP | Parameter | Fluorophore | Antibody | Company | Cat# | Concentration (ug/ul) | Volume to use (ul) |
|---|---|---|---|---|---|---|---|---|---|
| 405 Violet | 450/50 | 410 | BV421/DAPI/Pacific Blue/V450/S | BV421 | PD-L1 | BD | 568309 | 0.2 | 1 |
| | 525/50 | 505 | BV480/AmCyan/BV510/V500 | BV510 | Ly6G | Biolegend | 127633 | 0.2 | 1 |
| | 610/20 | 595 | BV605/FVS575V | SB600 | FceR1a | Invitrogen | 63-5898-82 | 0.2 | 1 |
| | 670/30 | 655 | BV650/SuperBright645 | BV650 | TLR4 | BD | 740615 | 0.2 | 1 |
| | 710/50 | 690 | BV711 | BV711 | MHCII | Biolegend | 107643 | 0.2 | 1 |
| | 780/60 | 750 | BV786 | BV786 | Siglec-F | BD | 740956 | 0.2 | 1 |
| 488 Blue | 530/30 | 505 | Alexa Fluor 488/FITC/GFP/BB515 | BB515 | CD11b | BD | 564454 | 0.2 | 1 |
| | 710/50 | 690 | RB705/PerCP-Cy5.5/BB700/Per | PerCP-eFluor71( | CD200R | Invitrogen | 46-5201-82 | 0.2 | 1 |
| 561 YG | 586/15 | 580 | PE | PE | MerTK | BD | 570196 | 0.2 | 1 |
| | 610/20 | 595 | PE-CF594/PE-Texas Red | PE-Dazzle594 | CD117 | Biolegend | 135128 | 0.2 | 1 |
| | 670/30 | 655 | PE-Cy5 | | | | | | |
| | 710/50 | 690 | PE-Cy5.5 | | | | | | |
| | 780/60 | 750 | PE-Cy7 | PE-Cy7 | CD11c | Biolegend | 117318 | 0.2 | 1 |
| 637 Red | 670/30 | 655 | Alexa Fluor 647/APC | APC | Ly6c | Biolegend | 128016 | 0.2 | 1 |
| | 710/50 | 690 | Alexa Fluor 700/**APC-R700**/R718 | R718 | CD45 | BD | 567075 | 0.2 | 1 |
| | 780/60 | 750 | APC-Cy7/APC-H7 | APC-Cy7 | FVS780 | BD | 565388 | 1 | 1 |

Lymphocyte Panel

| Laser | Filter | LP | Parameter | Fluorophore | Antibody | Company | Cat# | Concentration (ug/ul) | Volume to use (ul) |
|---|---|---|---|---|---|---|---|---|---|
| 405 Violet | 450/50 | 410 | BV421/DAPI/Pacific Blue/V450/S | BV421 | CD3e | Biolegend | 155617 | 0.2 | 0.625 |
| | 525/50 | 505 | BV480/AmCyan/BV510/V500 | BV510 | CCR6 | BD | 747832 | 0.2 | 0.125 |
| | 610/20 | 595 | BV605/FVS575V | BV605 | TRGV2 | BD | 742310 | 0.2 | 1.25 |
| | 670/30 | 655 | BV650/SuperBright645 | BV650 | TCR-g/d | Invitrogen | 416-5711-80 | 0.2 | 0.625 |
| | 710/50 | 690 | BV711 | BV711 | PD-1 | Biolegend | 135231 | 0.2 | 1.25 |
| | 780/60 | 750 | BV786 | BV786 | CD8a | Invitrogen | 417-0081-82 | 0.2 | 1.25 |
| 488 Blue | 530/30 | 505 | Alexa Fluor 488/FITC/GFP/BB515 | FITC | CD4 | Invitrogen | 11-0042-82 | 0.5 | 0.5 |
| | 710/50 | 690 | RB705/PerCP-Cy5.5/BB700/Per | PerCP-Cy5.5 | CCR4 | Biolegend | 131219 | 0.2 | 1.25 |
| 561 YG | 586/15 | 580 | PE | RY586 | B220 | BD | 756117 | 0.2 | 1.25 |
| | 610/20 | 595 | PE-CF594/PE-Texas Red | PE-CF594 | CD25 | Biolegend | 102048 | 0.2 | 1.25 |
| | 670/30 | 655 | PE-Cy5 | PE-Cy5 | NKP46 | Biolegend | 137647 | 0.2 | 1.25 |
| | 710/50 | 690 | PE-Cy5.5 | PE-Cy5.5 | Foxp3 | Invitrogen | 35-5773-82 | 0.2 | 1.25 |
| | 780/60 | 750 | PE-Cy7 | PE-Cy7 | CD44 | Biolegend | 103030 | 0.2 | 1.25 |
| 637 Red | 670/30 | 655 | Alexa Fluor 647/APC | APC | CD62L | Invitrogen | 17-0621-81 | 0.2 | 0.3 |
| | 710/50 | 690 | Alexa Fluor 700/**APC-R700**/R718 | R718 | CD45 | BD | 567075 | 0.2 | 1.25 |
| | 780/60 | 750 | APC-Cy7/APC-H7 | APC-Cy7 | FVS780 | BD | 565388 | 1 | 1 |

**SFig. 2. Flow cytometry panel design and information on antibodies used.**

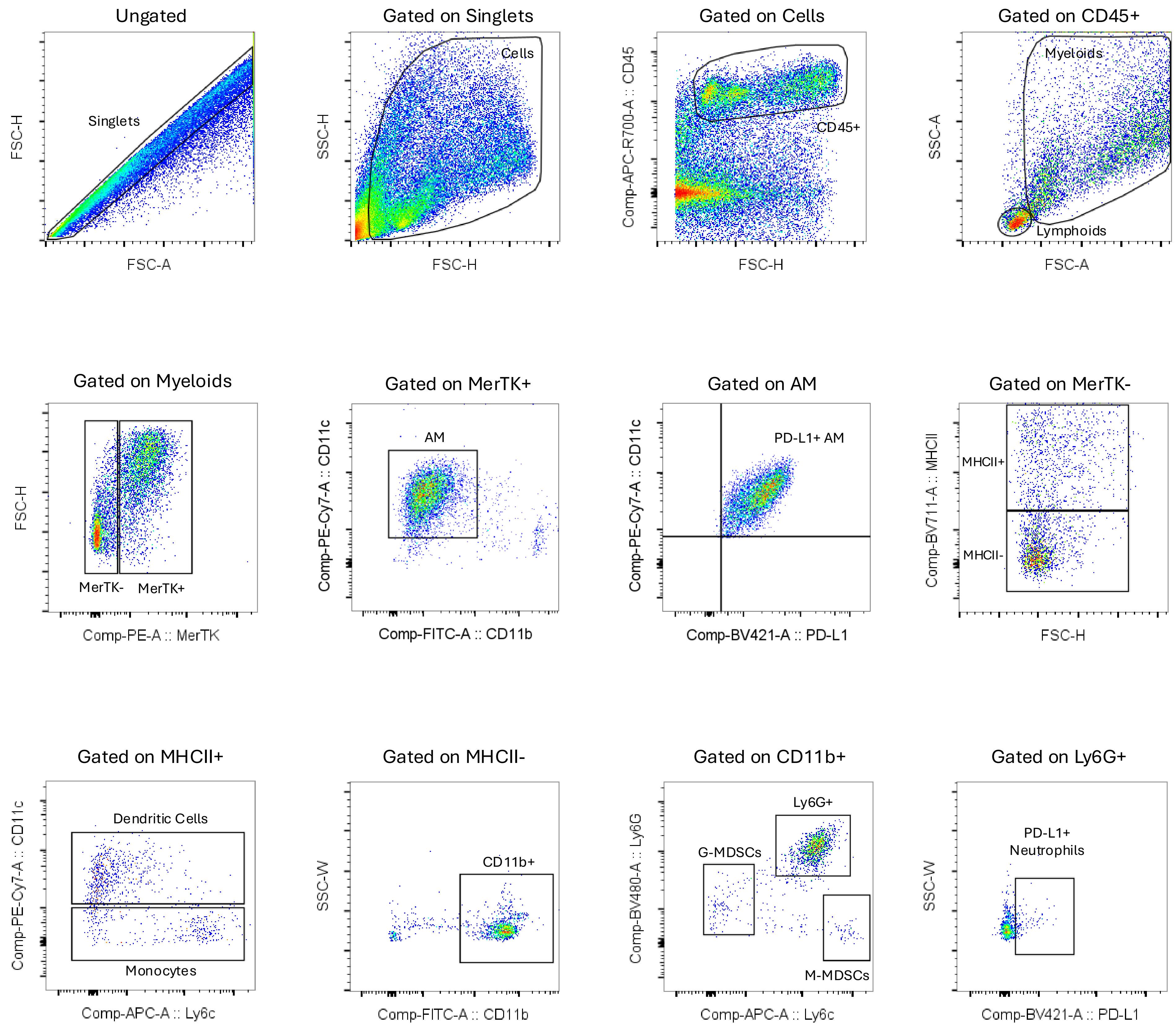


**SFig. 3. Gate strategy for myeloid cell flow cytometry analysis.**

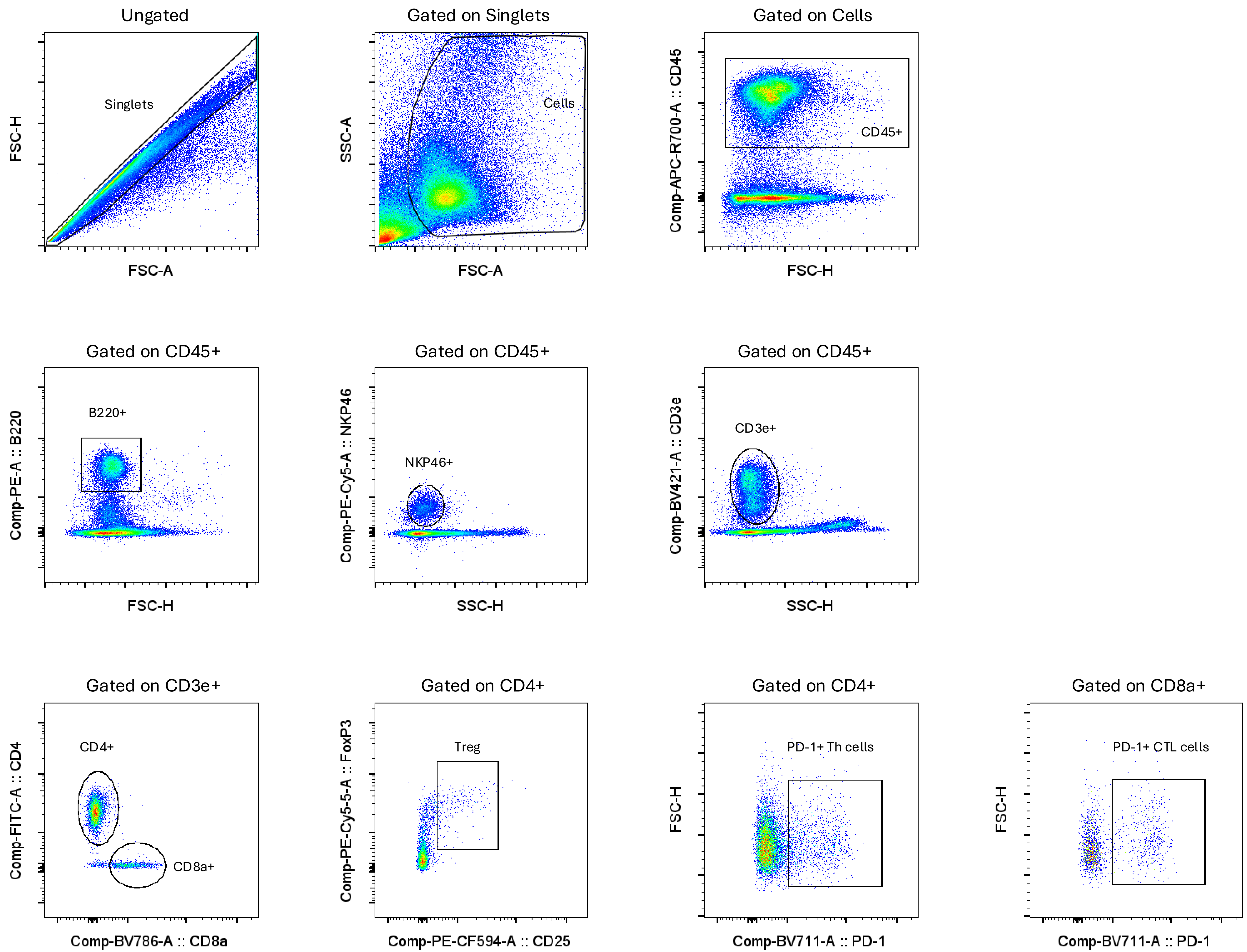


**SFig. 4. Gate strategy for lymphoid cell flow cytometry analysis.**
